# Endonasal surgery in the coronavirus era – Birmingham experience

**DOI:** 10.1017/S0022215120002364

**Published:** 2020-11-04

**Authors:** P P Naik, G Tsermoulas, A Paluzzi, L McClelland, S K Ahmed

**Affiliations:** 1 Department of ENT, University Hospital Birmingham, UK; 2 Department of Neurosurgery, University Hospital Birmingham, UK

**Keywords:** Skull Base, Endoscope, Nose, SARS-CoV, Pituitary Adenoma

## Abstract

**Background:**

The World Health Organization declared coronavirus disease 2019 a pandemic on 11th March 2020. There is concern regarding performing endonasal surgical procedures because of a high viral load in the nasopharynx. This paper describes our experience in conducting emergency and urgent endonasal operations during the peak of the coronavirus disease 2019 pandemic in the UK.

**Objectives:**

To show the outcome of endonasal surgery during the peak of the coronavirus disease 2019 pandemic and to assess the post-operative rate of nosocomial coronavirus disease 2019 infection.

**Methods:**

A retrospective cohort study was conducted of all patients who underwent high priority endoscopic nasal surgery or anterior skull base surgery between 23rd March and 15th June 2020 at University Hospitals Birmingham NHS Trust.

**Results:**

Twenty-four patients underwent endonasal surgery during the study period, 12 were males and 12 were females. There was no coronavirus-related morbidity in any patient.

**Conclusion:**

This observational study found that it is possible to safely undertake urgent endonasal surgery; the nosocomial risk of coronavirus disease 2019 can be mitigated with appropriate peri-operative precautions.

## Introduction

The World Health Organization declared coronavirus disease 2019 (Covid-19) a pandemic on 11th March 2020. The disease is caused by severe acute respiratory syndrome coronavirus 2 (SARS-CoV-2).^1^ The most common manifestations of infection include cough, fever, myalgia, a reduced sense of smell and taste, and dyspnoea.^2,3^

In addition to the transmission of infection to healthcare workers, there is concern about nosocomial Covid-19 infection. Research shows that there is a high viral load in the nasopharyngeal area.^4^ This creates a major concern when managing patients requiring transnasal endoscopic surgery, especially with the use of micro-debriders and drills, which cause aerosolisation of SARS-CoV-2.^5^ Global reports mention a high rate of infection amongst otolaryngologists.^6^ There is a general fear across the world, and this is extended to and forms a particular challenge for endoscopic rhinology surgery in the management of cancers and life-threatening conditions.

Trends show that there is likely to be a decline in the number of new Covid-19 cases; however, a further second or third peak has been predicted.^7^ In such a situation, it is necessary to maintain a balance between deferring and performing transnasal endoscopic surgical procedures. There are very limited data showing the impact and outcomes of high priority surgery during this pandemic. This article presents the outcomes of transnasal endoscopic operations performed during the peak of the Covid-19 pandemic in a tertiary care hospital in Birmingham, UK.

## Materials and methods

A retrospective study was conducted of 24 patients who underwent endonasal surgery, performed both by ENT surgeons and neurosurgeons, between 23rd March and 15th June 2020 at University Hospitals Birmingham NHS Trust, UK. This was the time frame when the UK declared a medical emergency and there was a peak in transmission rates.^8,9^

After considering the priority levels for surgery as per the UK guidelines, a total of 24 patients were prioritised as level 1 or 2 (Table 1),^10^ and underwent emergency or urgent endonasal surgery.^11^ One patient underwent surgery twice.

**Table 1.** Priority levels^13^

Level	Time frame for surgery
Level 1a	Within 24 hours
Level 1b	Within 72 hours
Level 2	Within 4 weeks
Level 3	Within 3 months

All patients were pre-operatively screened for Covid-19 infection with reverse transcription polymerase chain reaction testing, via the method described by national authorities.^11^ Apart from one emergency epistaxis case, all patients self-isolated for 14 days before and 14 days after surgery. All operations were performed with full personal protective equipment (PPE), which included: a long-sleeved, fluid-repellent disposable gown and gloves; a filtering face piece (FFP) code 2 or code 3 mask; a FFP3 respirator and loose-fitting respirator hood; and a visor. Patients were monitored for any symptoms of Covid-19 infection before discharge and two weeks post-surgery.

Informed consent was obtained from all patients, including the risk of acquiring Covid-19. All patients were managed according to the most up-to-date recommendations for surgery, following our hospital coronavirus priority operating plan. No separate ethical committee approval was sought as this was an observational study. Data were collected retrospectively, using medical records.

Regarding statistical analysis, continuous variables are presented as means; categorical variables are expressed as frequencies and percentages.

## Results

Twenty-four patients, 12 females and 12 males, underwent endonasal surgery during the study period, with 1 patient undergoing surgery twice. None of the patients had a history of recent travel. The patients’ age range was 19 to 84 years (mean, 53.4 years). The baseline patient characteristics are described in Table 2.

**Table 2.** Baseline characteristics

Characteristic	Value(s)
Age (mean (range); years)	53.4 years (19–84)
Males (*n*)	12
Females (*n*)	12

Thirteen of the patients had co-morbidities (Tables 3 and 4). Five patients had an immunocompromised state, with two undergoing chemotherapy, one undergoing radiotherapy for melanoma and two having uncontrolled diabetes mellitus.

**Table 3.** Other parameters

Parameter	Value(s)
Hospital stay (mean (range); days)*	4 (0–18)
Mortalities (*n*)	0
Morbidities related to Covid-19 (*n*)	0
Signs & symptoms of Covid-19 (*n*)	0
Immunosuppressive state (*n*)	5

*Two patients had significantly extended lengths of stay because of social or non-medical reasons, and were excluded from the length of stay calculation. Covid-19 = coronavirus disease 2019

**Table 4.** Co-morbidities in patients

Co-morbidity	Patients (*n*)
Hypothyroidism	1
Undergoing chemoradiotherapy for cancer	3
Arthritis; on immunotherapy	1
COPD or asthma	4
Hypertension	3
Diabetes mellitus	2
Neurofibromatosis type 2	1
Previous renal cancer	1

COPD = chronic obstructive pulmonary disease

All patients were discussed in a multidisciplinary meeting and were prioritised before surgery. They were all negative for Covid-19 at their pre-screening appointment. Six patients were priority level 1, with the remainder being priority level 2 (Table 5).

**Table 5.** Surgery type and priority level

Case number	Age (years)	Sex	Surgery name	Diagnosis	Co-morbidities	Priority level
1	56	M	Endoscopic trans-sphenoidal excision of pituitary lesion	Pituitary macroadenoma causing progressive visual loss	Hypothyroidism	Level 2
2	51	F	Endoscopic trans-sphenoidal excision of pituitary lesion	Pituitary macroadenoma causing progressive visual loss	–	Level 2
3	51	M	Endoscopic trans-sphenoidal excision of pituitary lesion	Pituitary macroadenoma causing progressive visual loss	–	Level 2
4	68	F	Endoscopic trans-sphenoidal excision of pituitary lesion	Pituitary macroadenoma causing progressive visual loss	Previous breast cancer, previous hysterectomy, stroke, hip replacement	Level 2
5, 6	60	F	Case 5: Endoscopic trans-sphenoidal excision of pituitary lesion Case 6: CSF leak repair after 4 weeks	Case 5: metastasis to pituitary causing progressive visual loss of the only eye Case 6: post-op CSF leak	Cases 5 & 6: arthritis, immunotherapy	Case 5: level 2 Case 6: level 1
7	69	F	Endoscopic trans-sphenoidal excision of pituitary lesion	Pituitary macroadenoma causing progressive visual loss	COPD, hypertension	Level 2
8	62	F	Sphenopalatine artery ligation	Uncontrolled epistaxis	Pulmonary embolism; on enoxaparin. Ongoing chemotherapy for breast cancer	Level 1b
9	76	M	Rhinectomy with endoscopic resection of nasal tumour	Squamous cell carcinoma of nose	–	Level 2
10	23	M	Endoscopic excision of clival chordoma	Clival chordoma	Asthma	Level 2
11	55	M	Draf III frontal drill out	Expansile polyps causing progressive visual deficit	Samter's triad	Level 2
12	76	F	Sphenoidotomy with mucocele drainage	Expansile mucocele causing progressive visual deficit	Asthma	Level 2
13	84	F	Extirpation of nasopharyngeal lesion for culture & histology	Skull base osteomyelitis due to diphtheria, with cranial nerve palsy	–	Level 2
14	77	F	Right FESS, debridement of orbital cellulitis with biopsy	Orbital cellulitis with chronic rhinosinusitis	Ongoing chemotherapy for leukaemia	Level 1
15	24	F	Endonasal repair of CSF leak	Spontaneous CSF leak	–	Level 1
16	69	F	Sphenopalatine artery ligation	Uncontrolled epistaxis	Previous kidney cancer & nephrectomy, hypertension	Level 1
17	24	M	Draf III, sphenoethmoidectomy & washout	Subdural abscess, frontal sinusitis	–	Level 2
18	20	M	Examination under anaesthesia of left orbit & paranasal cavities	Penetrating bullet wound to orbit	–	Level 1
19	19	F	Endoscopic trans-sphenoidal left optic decompression	Meningioma in orbital apex causing visual loss	Neurofibromatosis type 2	Level 2
20	84	M	Left sphenoethmoidectomy under image guidance	Fungal sinus ball causing optic neuropathy	Diabetes mellitus, hypertension	Level 2
21	61	M	Excision of pituitary macroadenoma under image guidance	Pituitary adenoma causing progressive visual field defect	–	Level 2
22	48	M	Draf III revision sphenoethmoidectomy under image guidance	Allergic fungal chronic rhinosinusitis	Diabetes mellitus	Level 2
23	35	F	Left transpterygoid repair of meningoencephalocele under image guidance with fluorescein lumbar puncture	Meningoencephalocele with CSF leak	–	Level 2
24	49	M	Excision of pituitary macroadenoma under image guidance	Pituitary adenoma causing progressive visual field defect	–	Level 2
25	34	M	Draf III frontosphenoethmoidectomy with left orbital transposition & drainage of fronto-orbital mucocele under image guidance	Left frontal mucocele causing progressive proptosis with chronic rhinosinusitis	–	Level 2

M = male; F = female; CSF = cerebrospinal fluid; post-op = post-operation; COPD = chronic obstructive pulmonary disease; FESS = functional endoscopic sinus surgery

One patient had an overnight stay in intensive care for observation, with a lumbar drain inserted. The average hospital stay was 4 days. During endoscopic surgery, debriders and drills were avoided where possible. All operations were undertaken in full PPE. Post-operatively, no patient had any signs or symptoms suggestive of Covid-19 infection.

At the time of discharge, patients were instructed to self-isolate for a period of 14 days and asked to contact the UK Covid-19 helpline if they became symptomatic.^12^ None of the patients were symptomatic at discharge from hospital or at their first telephone follow up at two weeks.

## Discussion

This article describes our experience of operating on nose and skull base lesions during the coronavirus pandemic on a high priority or urgent basis. The length of hospital stay was less than the median incubation period of SARS-CoV-2.^13,14^ Lei *et al*. identified a high rate of post-operative coronavirus infection.^15^ The most important aspect is the mortality associated with being operated on, and having Covid-19 or contracting it in immediate post-operative period.^16^ Although endoscopic nasal surgery is considered to be one of the most aerosol-generating procedures, our study showed a 0 per cent rate of infection with Covid-19.

Currently, there are sparse data on patients undergoing endoscopic nasal surgical procedures in the coronavirus era. Zhu and colleagues reported on a patient diagnosed with pituitary adenoma who unfortunately succumbed to Covid-19 and died of respiratory failure;^17^ however, the surgery was performed in a normal operating theatre, without additional precautions and PPE. In our study, the entire operating theatre team had taken appropriate measures, wearing full PPE.^18^

## Conclusion

We have demonstrated that urgent endonasal surgery may be undertaken, following careful patient selection and categorisation, in those prioritised as level 1 or 2, as long as there is a careful and thorough patient investigation.

*Primum non nocere* (first do no harm); in acknowledgment of the significant progression of disease processes if left unchecked, we suggest that surgical teams, as described in this paper, consider the option of endonasal surgery for urgent cases, rather than alternative external, more invasive approaches or no treatment. All patients should be prioritised for these surgical procedures, and where appropriate discussed in a multidisciplinary team setting, taking into account the disease process and the harm caused by not operating or offering an alternative, non-endonasal option.

Every hospital should establish their own local protocol based on the most up-to-date evidence, using national and international guidelines.^19,20^ Strict pre-operative and operative protocols should be followed for cases that cannot be deferred. Patients need to be thoroughly consented regarding the risks of surgery specific to the background rate of coronavirus in their locality and in relation to the anatomical region that is being operated on.

• All patients should be prioritised for endonasal surgery
• Prioritisation should consider the disease process and the harm caused by not operating or offering an alternative, non-endonasal option
• Every hospital should establish their own local protocol based on the most up-to-date evidence, using national and international guidelines
• Strict pre- and peri-operative protocols should be followed

In summary, our retrospective data of 24 patients who underwent 25 endonasal surgical procedures demonstrate a 0 per cent morbidity rate and mortality rate from operating endonasally in a controlled environment. We therefore recommend that patients with pathologies that demand urgent or immediate intervention not be denied the endonasal surgery route if this would have been the first-line approach before the Covid-19 pandemic.
